# Fatal Abdominal Compartment Syndrome Due to Constipation: A Case Report

**DOI:** 10.5811/cpcem.2021.7.53295

**Published:** 2022-01-04

**Authors:** Matthew M. Kongkatong, Malav M. Patel, Christopher Thom, James Moak

**Affiliations:** University of Virginia Medical Center, Department of Emergency Medicine, Charlottesville, Virginia

**Keywords:** abdominal compartment syndrome, constipation, case report, clozapine, surgical decompression

## Abstract

**Introduction:**

Abdominal compartment syndrome (ACS) is a rare condition in which increased intra-abdominal pressure causes multiorgan dysfunction through decreased perfusion. Causes of this condition are variable, and early recognition is critical for favorable patient outcomes. Measurement of bladder pressure is recommended for diagnosis.

**Case Report:**

A 64-year-old female on clozapine with a two-year history of chronic constipation presented to the emergency department in extremis with a protuberant abdomen. After resuscitative measures, computed tomography showed a dilated, stool-filled colon with a decompressed inferior vena cava and decreased perfusion. She died despite surgical decompression.

**Conclusion:**

Severe constipation is a rare cause of ACS, and there is a lack of evidence-based guidelines. Options for bedside decompression are limited. To reduce morbidity and mortality in this population, early recognition of ACS is imperative. Initial interventions should support hemodynamics and respiration. Definitive management is surgical decompression.

## INTRODUCTION

Abdominal compartment syndrome (ACS) is defined as organ dysfunction caused by an increase in intra-abdominal pressure greater than 20 millimeters of mercury (mm Hg). It is a rare condition that is more common in critically ill patients. The reported incidence of this condition ranges from 1–14% in studies of trauma patients and 12% in a study of patients with severe pancreatitis.^1^,^2^ High intra-abdominal pressure leads to compromised global and regional perfusion, resulting in life-threatening organ dysfunction. Abdominal compartment syndrome can cause reduction of respiratory volumes through mass effect on the diaphragm with resultant hypercarbia and hypoxemia. It can also cause metabolic acidosis, diastolic failure, increased intracranial pressure, oliguria, intracranial hypertension, and intestinal ischemia.^3^

The World Society of the Abdominal Compartment Syndrome (WSACS) categorizes this disorder by its underlying cause: decreased abdominal compliance (eg, burns); increased intra-abdominal contents (eg, hemoperitoneum, ascites); increased intraluminal contents (eg, intestinal volvulus, ileus, and constipation); capillary leak/fluid resuscitation; and miscellaneous causes such as obesity and peritonitis.^4^ Constipation, although a rare cause of ACS, is a common condition in older adults with a prevalence of 24–50%.^5^ Morbidity and mortality from ACS can be mitigated by timely surgical decompression with careful anticipation of reperfusion syndrome.

## CASE REPORT

A 64-year-old female with a history of chronic constipation and schizoaffective disorder on clozapine, risperidone, benztropine, and glycopyrrolate presented to the emergency department via emergency medical services (EMS) for a chief complaint of altered mental status concerning for acute stroke. The EMS personnel noted she was found by her boyfriend with slurred speech, left-sided weakness, and a severely distended abdomen. On first assessment she was obtunded and in shock with a heart rate of 131 beats per minute and blood pressure of 60/40 mm Hg. Oxygen saturation could not be obtained via skin probe, and respirations were 31 breaths per minute. She was hypothermic at 34.8 degrees Celsius. Her abdomen was grossly distended and tense with dullness to percussion in all quadrants. The patient was unable to provide any history but review of the electronic health record showed that she had a history of severe constipation and had seen her primary physician two weeks prior for the same complaint.

Femoral central access was obtained after three attempts with the first two attempts complicated by lack of blood return from the catheter after passage over the guidewire, which was misinterpreted as line misplacement. Point-of-care ultrasound demonstrated normal compression of the common femoral veins at both sites. Initial point-of-care blood testing showed a severe metabolic acidosis with pH of 6.36 and a lactic acid of 21.9 millimoles per liter (mmol/L) (reference range: 0.5–1.0 mmol/L) as well as profound anemia with a hemoglobin level of 3.7 grams per deciliter (g/dL) (reference range: 12.0–15.5 g/dL). Her creatinine level measured at 1.3 milligrams per deciliter (mg/dL), (reference range: 0.6–1.1 mg/dL) which was increased from her baseline of 0.9 mg/dL.

In total, she received two liters of 0.9% saline, three units of packed red cells, and two units of fresh frozen plasma without improvement of hemodynamics. A norepinephrine infusion was then initiated with improvement of blood pressure to 128/78 mm Hg. After improvement of hemodynamics the patient was intubated. Cefepime and metronidazole were given for presumed intra-abdominal sepsis. She also received a total of 250 milliliters (mL) of 8.4% sodium bicarbonate with improvement of her acidosis from pH 6.36 to pH 7.33. Her hyperkalemia of 7.8 mmol/L (reference range: 3.4–4.8 mmol/L) improved with fluids and sodium bicarbonate to 5.2 mmol/L. A forced-air warming device was applied to the patient after intubation.

**CPC-EM Capsule**

What do we already know about this clinical entity?

*Abdominal compartment syndrome (ACS) is a rare but life-threatening condition that requires prompt identification and aggressive management*.

What makes this presentation of disease reportable

*Our patient presented with ACS due to constipation, which is often considered a benign condition. Constipation as the cause of ACS has not been commonly reported*.

What is the major learning point?

*Untreated constipation can have serious consequences. Attention should be paid to constipation-causing medications in patients presenting for this complaint*.

How might this improve emergency medicine practice?

*Familiarization with the presentations of ACS, evaluation, and treatment of underlying causes may improve care of patients with this condition*.

**Image 1:**
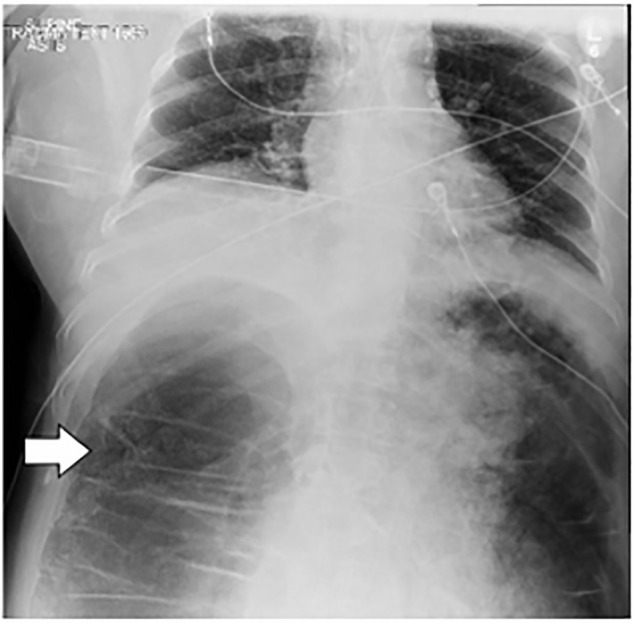
Supine chest radiograph after intubation demonstrating extremely dilated colon (arrow).

**Image 2:**
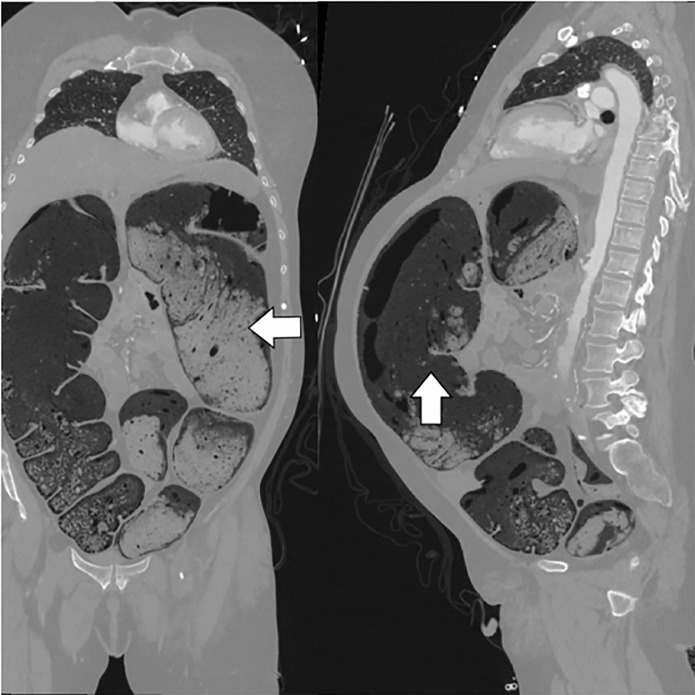
Coronal (left) and sagittal (right) reconstructions of the computed tomography angiogram of the chest, abdomen, and pelvis showing stool impaction causing massive colonic distention (arrows). The image window is set to lung to better visualize the stool within the colon.

**Image 3:**
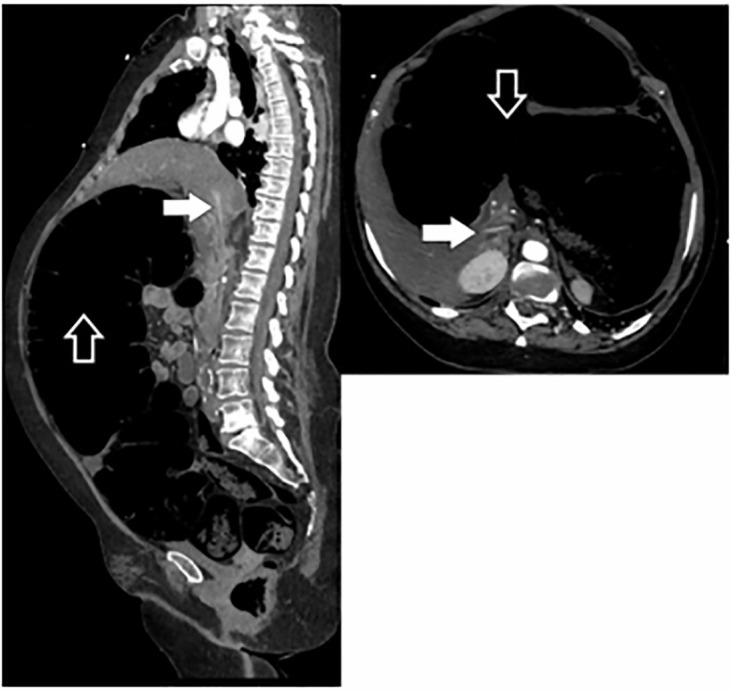
Sagittal (left) and axial (right) windows of the computed tomography angiogram of the chest, abdomen, and pelvis. The inferior vena cava is compressed (white arrows) by the massively dilated colon (black arrows).

**Image 4:**
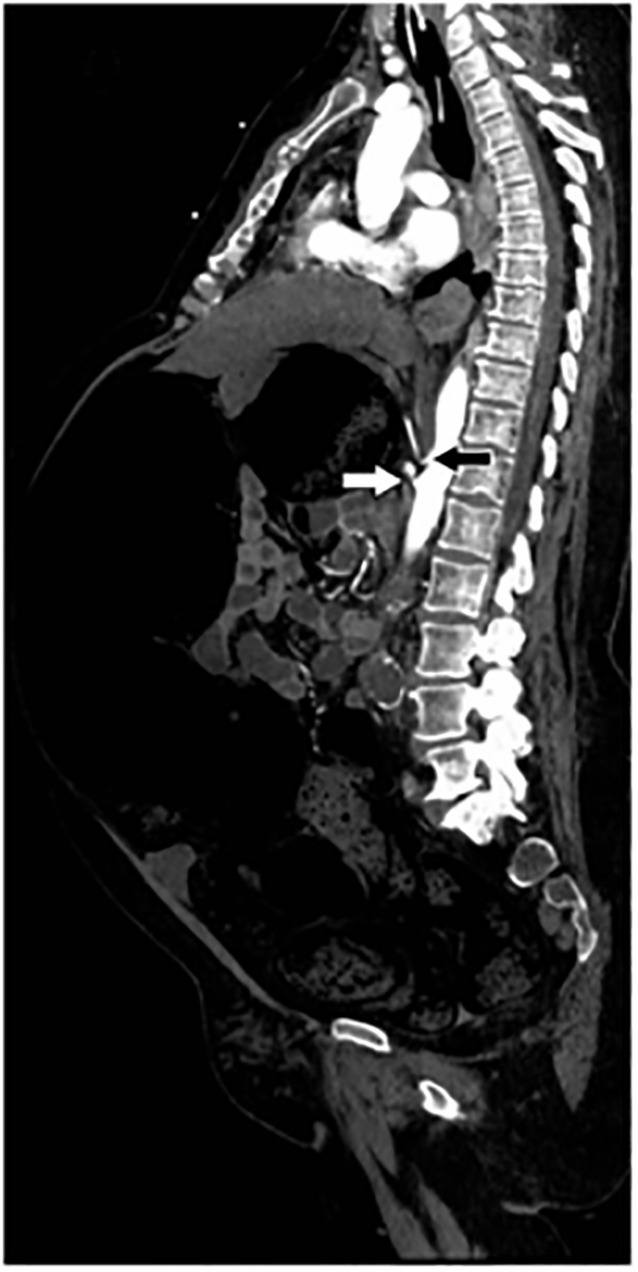
Sagittal window of the computed tomography angiogram of the chest, abdomen, and pelvis demonstrating ostial stenosis of the celiac artery (black arrow) and superior mesenteric artery (white arrow).

Point-of-care ultrasound revealed no intra-abdominal fluid. Supine chest radiography (Image 1) performed after intubation showed low lung volumes with a massively dilated colon. General surgery was consulted for acute abdomen, and additional imaging was obtained once her hemodynamics stabilized. Computed tomography (CT) of the head showed no intracranial hemorrhage or ischemic stroke. However, CT angiography of the chest, abdomen and pelvis was remarkable for a markedly dilated, stool-filled colon with diffuse pneumatosis (Image 2); no transition point was identified. The inferior vena cava was compressed (Image 3), and there was ostial stenosis of the celiac trunk and superior mesenteric artery (Image 4). Impaired perfusion of both kidneys was noted. Although intra-abdominal pressure was not transduced, the evidence of organ dysfunction with a tense abdomen and CT findings led to the diagnosis of ACS.

General surgery took the patient to the operating room emergently for decompressive laparotomy and total colectomy. Upon opening her fascia, the ischemic-appearing colon explosively decompressed, sending stool across the room. She subsequently experienced a wide complex tachycardia in the setting of a potassium of greater than 10 mmol/L. After multiple rounds of medications per Advanced Cardiac Life Support protocol, she was pronounced dead due to severe reperfusion injury. The surgeons noted a loop of sigmoid colon trapped by a band of tissue in the pelvis, when removing the colon for pathology assessment.

## DISCUSSION

Our patient presented in profound shock due to constipation-induced ACS and died despite aggressive management. Abdominal compartment syndrome is a rare condition with a scarcity of evidenced-based guidelines. Abdominal compartment syndrome can be caused by diminished abdominal wall compliance caused by abdominal surgery, major trauma, ileus, and burns. Increased intra-abdominal contents including abscess, ascites, hemoperitoneum, masses or air should also be considered. Furthermore, capillary leak that occurs in conditions such as sepsis, pancreatitis, acidosis, hypothermia, and coagulopathy can lead to this condition. If there is suspicion for intra-abdominal hypertension (IAH), then intra-abdominal pressure should be measured. The WSACS recommends trans-bladder measurement. This is done by attaching a pressure transducer to the sampling port of an indwelling urinary catheter via a 3-way stopcock, instilling 25 mL of saline into the bladder, then clamping the catheter distal to the sampling port.^3^ Alternative apparatuses using water manometry such as those found in lumbar puncture kits have also been described.^6^ When IAH is identified, point-of-care ultrasound and CT can help identify the underlying cause.^4^

Initial management is aimed at supporting organ perfusion. In obtunded patients with poor gas exchange, early intubation can optimize ventilation and alveolar recruitment. In addition to hemodynamic support with crystalloid, colloid, or vasopressors, consider evacuating intraluminal contents by nasogastric and/or rectal tube. Bedside paracentesis can be performed for large volume ascites, and emergent decompression of pneumoperitoneum can be considered.^7^ Decreased abdominal wall compliance can be addressed by optimizing sedation or paralysis. Burn escharotomy may also be performed. Placing the patient in reverse Trendelenburg position can also help with this by off-loading pressure from the inferior vena cava, as well as reducing mass effect on the diaphragm to improve lung compliance.^4^ Reperfusion injury should be anticipated after decompressive interventions.

Our patient’s presentation was caused by severe constipation. Our literature review identified one case of ACS in an adult caused by constipation likely secondary to neurogenic bowel^8^ and two cases thought to be due to clozapine use.^9^ Her longstanding constipation was likely due to her medication regimen of clozapine, risperidone, and the anticholinergic medications glycopyrrolate and benztropine. Clozapine is effective for treatment-resistant schizophrenia but is prescribed uncommonly (66.7 per 100,000 persons in the 2010 Medicaid database).^10^ In a meta-analysis from 2016, constipation was reported in 31% of patients taking clozapine, nearly three times more than patients taking other antipsychotics.^11^ A recent study of reports concerning clozapine to the Australian Therapeutic Goods Administration and New Zealand Pharmacovigilance Center found an 18% mortality rate over 22 years in patients with gastrointestinal adverse reactions serious enough to require hospitalization or surgical intervention. The same study reported data from a World Health Organization registry with a case fatality rate of 13% for the complaint of constipation. Case fatality rates were higher when sequela of constipation such as intestinal obstruction (25%) and intestinal ischemia (68%) were reported.^12^ It is unknown whether ACS was diagnosed in any of those patients. Risperidone, glycopyrrolate, and benztropine have also been implicated as causes for constipation.^13^–^15^

## CONCLUSION

Abdominal compartment syndrome causes multiorgan dysfunction and can result in shock and death despite timely recognition and aggressive management as in our patient. Emergency physicians should be suspicious for this condition in patients with a distended abdomen and abnormal vital signs. Interventions should be aimed at correcting metabolic, respiratory, and cardiovascular derangements as well as decompressing the peritoneal cavity via nasogastric/rectal decompression, paracentesis, or burn escharotomy as clinically indicated. The definitive treatment of ACS is surgical decompression. This case report demonstrates that severe constipation can be a fatal cause of ACS necessitating surgical intervention. It is an important reminder to the emergency physician that even seemingly benign conditions such as constipation can lead to severe complications if left unattended. It also underscores the importance of recognizing and treating chronic constipation, with particular attention to those patients on medications such as clozapine that are known to affect gastrointestinal motility.
